# Mass casualty modelling: a spatial tool to support triage decision making

**DOI:** 10.1186/1476-072X-10-40

**Published:** 2011-06-10

**Authors:** Ofer Amram, Nadine Schuurman, Syed M Hameed

**Affiliations:** 1 Geography Department, Simon Fraser University, 8888 University Drive, Burnaby, B.C., V5A 1S6, Canada; 2 Geography Department, Simon Fraser University, Geography Department, Simon Fraser University, 8888 University Drive, Burnaby, B.C., V5A 1S6, Canada; 3 University of British Columbia, General Surgery Residency Program, 2329 West Mall Vancouver, B.C., V6T 1Z4, Canada

## Abstract

**Background:**

During a mass casualty incident, evacuation of patients to the appropriate health care facility is critical to survival. Despite this, no existing system provides the evidence required to make informed evacuation decisions from the scene of the incident. To mitigate this absence and enable more informed decision making, a web based spatial decision support system (SDSS) was developed. This system supports decision making by providing data regarding hospital proximity, capacity, and treatment specializations to decision makers at the scene of the incident.

**Methods:**

This web-based SDSS utilizes pre-calculated driving times to estimate the actual driving time to each hospital within the inclusive trauma system of the large metropolitan region within which it is situated. In calculating and displaying its results, the model incorporates both road network and hospital data (e.g. capacity, treatment specialties, etc.), and produces results in a matter of seconds, as is required in a MCI situation. In addition, its application interface allows the user to map the incident location and assists in the execution of triage decisions.

**Results:**

Upon running the model, driving time from the MCI location to the surrounding hospitals is quickly displayed alongside information regarding hospital capacity and capability, thereby assisting the user in the decision-making process.

**Conclusions:**

The use of SDSS in the prioritization of MCI evacuation decision making is potentially valuable in cases of mass casualty. The key to this model is the utilization of pre-calculated driving times from each hospital in the region to each point on the road network. The incorporation of real-time traffic and hospital capacity data would further improve this model.

## Introduction

On July 7^th^, 2005, a series of terrorist attacks shook the London transit system [1]. Four bombs exploded almost simultaneously in a coordinated attack that left the city in a state of chaos [2]. Based on the sheer number of casualties, the incident has been described as the largest mass casualty incident in the United Kingdom since World War Two. Altogether, 775 people were injured in the attack, of which 56 died and 55 were critically injured. Casualties were divided amongst six hospitals (inclusive) within the city, based on hospital proximity, capacity and capability [2].

The following paper describes a spatial decision support system (SDSS) intended to help determine where best to evacuate patients during a mass casualty incident (MCI) of this type.

Mass casualty incidents are those that, by the sheer number and severity of casualties, overwhelm the health care capacity within a given community [3-5]. This definition emphasizes the crucial role played by triage and trauma centers in maximizing capacity during a mass casualty incident [6]. A concept that originated on the battlefield, triage, meaning 'to sort' in French, is one of the critical factors in the effective management of mass casualty incidents and refers to the process of prioritizing medical care based on the medical condition of the patient [7-9].

Intended to simplify and make evidence-based decisions concerning the evacuation of critically injured patients from an MCI location, this SDSS provides the information required by emergency service personnel at MCI location to make decisions in what is typically, a highly stressful and often chaotic situation. In addition to providing, within a matter of seconds, critical information describing hospital driving time/proximity, trauma level and bed capacity, the model is also useful within a planning context. For example, the model can be used to examine proposed locations for large scale events, conferences, etc. in relation to health care facilities or to help to determine where to position a mobile health facility in relation to the event.

Spatial models have been used within emergency services (EMS) for some time. Location allocation models, for example, are used to position facilities so as to optimize services to customers. In EMS, such models are focused on the optimization of ambulance locations in order to maximize coverage [10-13]. These models have evolved from the simple static models first developed 30 years ago to incorporate dynamic circumstantial changes. For example, such models can determine how best to fill the gap in coverage that is created when an ambulance within a particular geographical catchment is dispatched. In recent years, there have been a handful of attempts to optimize ambulance response times using models that incorporate dynamic traffic changes [14-16]. Advances in computer technologies that support decision making have made this process easier.

Combining geographic information systems (GIS) with decision support systems (DSS), Spatial Decision Support Systems (SDSS) were first introduced in the mid 1980's [17,18]. Decision support systems consist of distinct data management, model and interface components. Spatial Decision Support Systems add the visualization of spatial attributes, while Geographic Information Systems enable spatial data to be stored, manipulated and displayed. SDSS provide the ability to solve and simplify complex spatially-oriented problems [19-22]. In recent years a new kind of SDSS has emerged; one that relies on the web as a platform for interaction with the user. Made possible by increases in the speed of data transfer between client and server computers, web based SDSS enable greater information sharing and heightened use by non experts [23]. Web based SDSS also allow for the building of customized GIS applications that can be used with a remote server. These applications are platform independent and therefore more widely accessible. They are also purpose built, with tailored commands and functions making the application simpler to operate and understand than a full blown desktop application [24-26]. To date, no known modelling of MCI evacuation priorities has been undertaken and no emergency service models have been created to aid in evacuation prioritization. While there have been a few attempts to model optimal EMS routing to the scene of an incident, there was only one known attempt to model the return [15,16]. Drawing inspiration from the EMS models described above, the SDSS proposed within this paper also incorporates the use of GIS in the calculation of road network driving times.

## Methods

### Data

Two sets of data were used in constructing this model: road network data and hospital location data. The road data for metro Vancouver, obtained through GIS Innovations [27], is highly suitable for calculating travel time as it incorporates both speed limits and travel impedances (i.e. stop signs, traffic lights, etc.) which, in turn, allow for accurate travel time calculation. The data also provides the ability to control travel and impedance times. This is important, as travel times for an ambulance will differ from that of a regular vehicle. The fact that this data enables control of such variables heightens the accuracy of the results. The road network dataset used in this study excluded back roads and logging roads in order to focus on the more populated sections of the study area. Excluding these smaller roads also helped to reduce the database size.

The second set of data utilized in this study is comprised of the locations of participating hospitals within the metro Vancouver region. In addition to geocoded hospital locations, the hospital dataset also attaches attributes describing the hospital's capacity to receive patients in the case of a mass casualty incident and the type of treatment a given hospital is able to provide (Table 1). For trauma services, the range of services includes ICU, neurosurgery, orthopedics and plastic surgery. The hospitals are represented as a set of GIS point features and are geocoded as close to the main emergency room access as possible. As large hospitals can span several street blocks, geocoding the ER location rather than the hospital centroid can produce more accurate driving time results.

**Table 1:** Trauma center designation in Canada [28]

Level of Care	
**1**	Central role in the provincial trauma system, and the majority of tertiary/quaternary major trauma care in the system. Academic leadership, teaching, research program
**2**	Provides care for major trauma, Some trauma training and outreach programs. Similar to level 1 without academic and research program
**3**	Provides initial care for major trauma patients and transfer patients in needs of complex careto level 1 and 2 trauma centers
**4**	Major urban hospital with a nearby major trauma center (level 1-3). Does large volume of secondary trauma care. Bypass and triage protocols are in place diverting major trauma patients to level 1 and 2 centers.

In order to obtain results in a more immediate fashion, this model utilized pre-calculated driving times from each location on the road network to each hospital in the study area. Before pre-calculating the driving times, the data first had to be discretized to a length which would minimize the effect on actual driving time calculation. By restricting the length of the discretized road segments to a maximum of 200 m, it was determined that accurate driving times could be achieved without negatively affecting either the results or the size of the road dataset. The same road data used for the driving time calculation was also used to create the road segments. Close examination of the GIS Innovations [27] data indicated that the road segments within the data varied drastically in length, with segments both much smaller and much larger than 200 m. After several experiments, it was found that leaving all road segments below 200 m unchanged and subdividing all road segments larger than 200 m to the 200 m maximum worked most effectively. The 200 m street segments provided accurate driving times while also keeping the size of the database manageable. The resulting dataset contains road segments of varying lengths, with no segment larger than the 200 m maximum.

**Figure 1:**
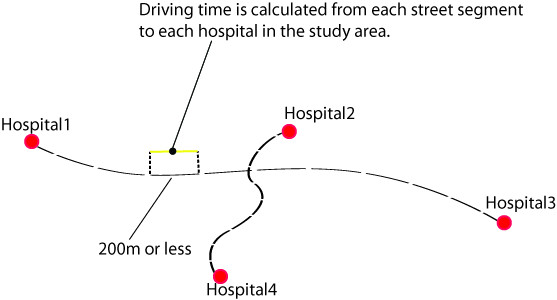
**Shows the method of pre calculating driving times to each hospital in the study area**. The road network is divided into segments 200 m or less in length. Driving time to each hospital is then calculated from each road segment in the study area.

In order to calculate driving time from each road segment to each hospital, each road segment was converted into a centroid. The ODMatrix function within ESRI ArcGIS network analyst was then used to calculate driving time to each hospital. The ODMatrix function calculates the shortest driving time from each point of origin to each destination on the road network producing a 'drivingTime' table which contains a unique ID for each centroid plus the driving time in minutes to each hospital [29]. In order to attain greater accuracy, an impedance time value was obtained from experienced paramedics and assigned to both stop signs (5 second) and traffic lights (10 seconds). The table also produces a hospital unique ID for each destination hospital. Once this table was created, the centroid ID was reassigned to its road segment so that the user could click on the road segment and retrieve its unique ID (Figure 1). The road data set consisted of a road segment shapefile within which each segment was related to the driving time table through a one-to-many relationship.

The final step in the data preparation was to create the hospital data list. This was a relatively simple task, as all the information was readily available, the locations were known and only a relatively small number of hospitals were involved in the study. As part of the data preparation, each hospital was given a unique ID corresponding to the driving time table with a many-to-one relationship.

### Model Construction

The construction of the model was divided into two distinct parts: creation of the mapping interface (the SDSS) and creation of a mechanism to analyze and process the data (model). The mapping interface was designed to allow the user to zoom to a location and to click on a road segment and insert a location into the map. In order to facilitate this, the 200 m segmented road data was first uploaded into ArcGIS server. A block of code was then written to allow users to click on a road segment, insert an MCI location and retrieve the unique ID of the road segment. Once retrieved, the unique ID is used to obtain the driving time to each hospital from the pre calculated driving time table. This portion of the model was constructed using ArcGIS server API, as it provides a rich set of functionalities and tools to interact with the road data and allow developers to build complex web-based mapping applications.

**Figure 2:**
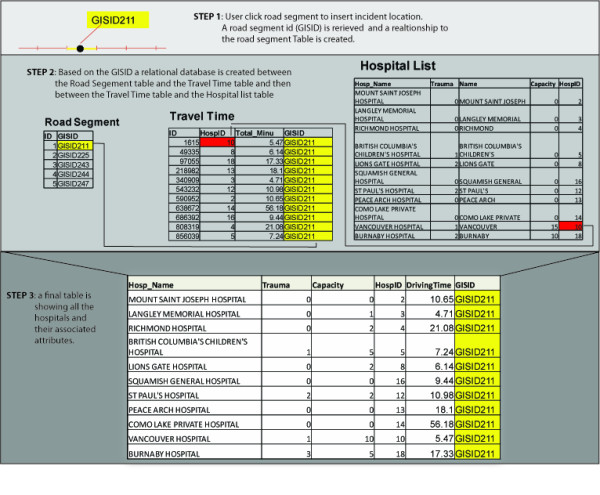
**Illustrates creation of hospital table and its associated attributes**.

The second aspect of constructing the model involved creating a mechanism to join the unique ID from each road segment to the pre calculated driving time table, establishing a database relationship between the driving time table and the hospital table, and analyzing and visualizing the resulting data (Figure 2). For this purpose, VB.NET[30] was utilized as the server side scripting language while javascript was used as the client side scripting language. VB.NET[30] enables database interaction and provides a set of decision making tools for the analysis and visualization of results using tables and graphs. More specifically, VB.NET[30] is used to compile the data and display the results based on the user's input. The entire model, including mapping and analysis, was built in Visual Web Developer (VWD) 2008 express edition [31].

## Results

**Figure 3:**
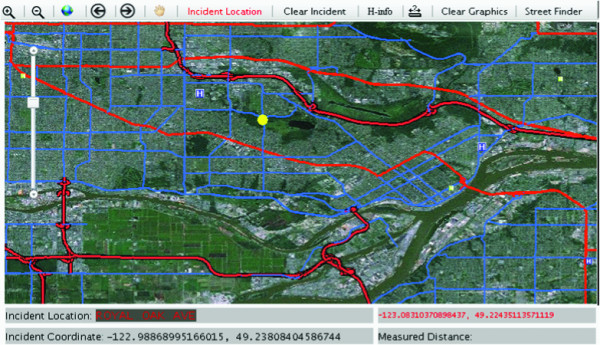
**A digital map indicates the location of the MCI and surrounding hospitals**.

The database becomes active when the user enters the web site and a connection to the hospital data table is established as the page loads. Once this takes place, the user can modify the default hospital capacity and determine which hospitals should be included in the analysis. The user then needs to insert the MCI location into a high resolution map (Figure 3) and enter additional information like the incident reference location. After an MCI location is inserted into the map, the model is ready to be executed. Upon running the model, a new results page opens listing each hospital, its associated attributes and its driving time from the MCI location. The results page provides a visual representation of the analysis, using both tables and graphs.

**Figure 4:**
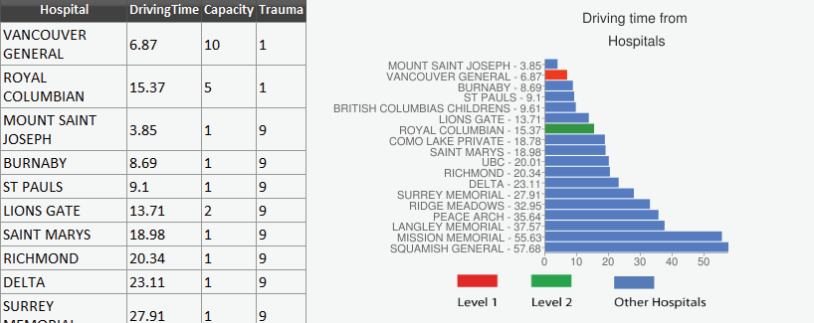
**Shows hospital driving times created during a simulation**. The table provides information regarding the proximity of hospitals to the MCI, their capacity and trauma level. Trauma level 1 hospitals are preferred when located in close proximity to the MCI location. However, in cases where Trauma level 1 hospitals are full or busy, the nearest non-trauma hospital will be utilized.

In order to test the model, a simulated MCI was created within the study area, using casualty counts from the 2005 London bombings. Using the King's Cross counts, where 10 critically injured patients were evacuated, an incident location was inserted at Broadway sky train station, one of Vancouver's busiest train stations. Figure 4 shows the results page produced by the simulation. Driving times to each of the hospitals in the study area are shown along with hospital capacity and trauma level. The results indicate that patients should be distributed between Vancouver General Hospital and Royal Columbian Hospital. In addition to driving times to trauma hospitals, the proximity to the nearest non-trauma hospital (depicted as trauma level 9) is also important as it provide an option in cases where the trauma hospitals become overloaded.

### Driving Time Validation

**Figure 5:**
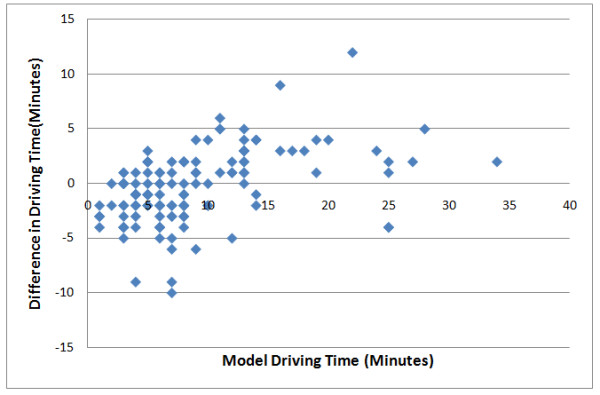
**Shows how actual ambulance driving times deviate from driving times within the model**.

Figure 5 and table 2 illustrate differences between the model's driving times and actual ambulance driving times collected from two ambulance stations within the metro Vancouver area. The driving times that were collected were for critically injured patients only. One ambulance station was located within an urban setting while the other was located in suburban Metro Vancouver. After filtering the data to show only trips occurring between 7 pm and 7 am, and 12 to 3 pm, the 132 ambulance trips showed larger variability in the ambulance driving time compared with the model driving time. The graph shows that the model underestimates and overestimates driving time in both long and short ambulance trips. There are several reasons that this may have occurred. First, the model driving times were rounded to the minute in order to be able to compare them to ambulance driving times (ambulance results were logged in minutes). Second, ambulance driving time records were taken from the ambulance paper log and there is no way to track at which point in the ambulance trip the start and end time of the trips were entered into the paper sheet. Both of these issues may drastically affect the results, particularly when the trip time is short. These unavoidable inconsistencies may partially explain the variability scatter in the graph in Figure 5.

**Table 2:** Shows comparison between model driving time and actual ambulance driving time for nine ambulance trips that had the same origin and destination

Origin	Destination	Model Time(Min)	Ambulance Time(Min)
SMH	RCH	13	10
SMH	RCH	13	12
SMH	RCH	13	11
SMH	RCH	13	12
SMH	RCH	13	8
SMH	RCH	13	27
SMH	RCH	13	10
SMH	RCH	13	13
SMH	RCH	13	9
SMH	RCH	13	10
SMH	RCH	13	11

The table below shows nine incidents where ambulance trips started and ended in exactly the same location. In this case, patients were being transferred from a non-trauma hospital to a major trauma hospital. The model time calculation was 13 minutes while most of the actual ambulance driving time ranges from 8 to 13 minutes with one trip as an outlier at 27 minutes. The table results illustrate the variability between trips from and to the same locations. The results from the table illustrate the relatively limited variability of ambulance driving time compared to our model.

## Conclusion

The response to a MCI must be both swift and precise if it is to be effective. As a result, dynamic decision making is of critical importance [32]. To be useful in this context, MCI modelling must produce results within an extremely short time frame. Although the proposed model provides the basic information required for evidence-based decision making, improvements can still be made, particularly in regard to the provision of real time hospital capacity and traffic data. Real time hospital capacity can be obtained by creating a utility that will enable hospitals to update capacity in the hospital database as soon as a mass casualty is declared. The model can then connect to the hospital database to retrieve the capacity. In addition, the model allows updates in hospital capacity as patients are evacuated from the scene of the incident to a given hospital. Unfortunately, incorporating real time traffic data is more complicated, as to do so would significantly extend the time required for computer data processing [16,33]. Although the model described in this study was able to avoid significant processing delays by utilizing pre-calculated driving times from each location on the road network to each hospital in the study area, the use of pre-calculated driving times also introduces some limitations. It does not, for example, allow for the input of travel impedances, like street closure as a result of the MCI, like bridge closures, or construction, into the calculation. Table 2, which compares model travel times with actual ambulance travel times, highlights the need to implement travel time calculations in real time while also incorporating real time traffic data. Out of the nine identical ambulance trips that were recorded, one trip clearly took much longer than the others. While the reason for this particular delay is unknown, a real time traffic data and driving time calculation might have suggested a different route if a traffic problem were the cause.

During an MCI, decisions regarding the evacuation of patients are based on an evaluation of injury type and severity, in relation to hospital proximity and capacity. The web based model proposed within this study is intended to provide evidence-based hospital and driving time information in a timely manner to assist in the onsite management of MCI incidents.

## Competing interests

The authors declare that they have no competing interests.

## Authors' contributions

OA created the MCI user interface, conducted all travel time modelling and verification and drafted the manuscript. NS contributed to the project conceptualization, interface refinement and writing and editing of the manuscript. MH was involved in the project conceptualization and ambulance cross-checking. All authors read and approved the final manuscript.
